# Inhibition of Alternative Cancer Cell Metabolism of EGFR Mutated Non-Small Cell Lung Cancer Serves as a Potential Therapeutic Strategy

**DOI:** 10.3390/cancers12010181

**Published:** 2020-01-10

**Authors:** Chung-Yu Huang, Li-Han Hsu, Chung-Yeh Chen, Gee-Chen Chang, Hui-Wen Chang, Yi-Mei Hung, Ko-Jiunn Liu, Shu-Huei Kao

**Affiliations:** 1 Ph.D. Program in Medical Biotechnology, College of Medical Science and Technology, Taipei Medical University, Taipei 11031, Taiwan; d609105006@tmu.edu.tw (C.-Y.H.); lhhsu@kfsyscc.org (L.-H.H.); g160090005@tmu.edu.tw (H.-W.C.); 2 School of Medical Laboratory Science and Biotechnology, College of Medical Science and Technology, Taipei Medical University, Taipei 11031, Taiwan; ian973921@gmail.com (C.-Y.C.); kojiunn@nhri.org.tw (K.-J.L.); 3 Division of Pulmonary and Critical Care Medicine, Sun Yat-Sen Cancer Center, Taipei 11259, Taiwan; 4 Faculty of Medicine, School of Medicine, National Yang-Ming University, Taipei 11221, Taiwan; august@vghtc.gov.tw; 5 Division of Chest Medicine, Department of Internal Medicine, Taichung Veterans General Hospital, Taichung 40705, Taiwan; 6 Institute of Biomedical Sciences, National Chung-Hsing University, Taichung 40227, Taiwan; 7 Department of Medical Laboratory, Taipei Medical University Hospital, Taipei 11031, Taiwan; 8 National Institute of Cancer Research, National Health Research Institutes, Zhunan, Miaoli 35035, Taiwan; yimei@nhri.org.tw; 9 Institute of Clinical Pharmacy and Pharmaceutical Sciences, National Cheng Kung University, Tainan 70101, Taiwan

**Keywords:** NSCLC, gefitinib-resistant, EGFR, alternative metabolism, monocarboxylate transporter 1

## Abstract

Targeted therapy is an efficient treatment for patients with epidermal growth factor receptor (EGFR) mutations in non-small cell lung cancer (NSCLC). Therapeutic resistance invariably occurs in NSCLC patients. Many studies have focused on drug resistance mechanisms, but only a few have addressed the metabolic flexibility in drug-resistant NSCLC. In the present study, we found that during the developing resistance to tyrosine kinase inhibitor (TKI), TKI-resistant NSCLC cells acquired metabolic flexibility in that they switched from dependence on glycolysis to oxidative phosphorylation by substantially increasing the activity of the mitochondria. Concurrently, we found the predominant expression of monocarboxylate transporter 1 (MCT-1) in the TKI-resistant NSCLC cells was strongly increased in those cells that oxidized lactate. Thus, we hypothesized that inhibiting MCT-1 could represent a novel treatment strategy. We treated cells with the MCT-1 inhibitor AZD3965. We found a significant decrease in cell proliferation and cell motility in TKI-sensitive and TKI-resistant cells. Taken together, these results demonstrated that gefitinib-resistant NSCLC cells harbored higher mitochondrial bioenergetics and MCT-1 expression. These results implied that targeting mitochondrial oxidative phosphorylation proteins or MCT-1 could serve as potential treatments for both TKI-sensitive and –resistant non-small cell lung cancer.

## 1. Introduction

Lung cancer is the most commonly diagnosed cancer in the world (1.8 million new cases in 2012) and currently represents the most common cancer in Taiwan [1,2]. The two histological types of lung cancer are small cell lung cancer and non-small cell lung cancer (NSCLC). Epidermal growth factor receptor (EGFR) mutations play a crucial role in NSCLC. In Taiwan, EGFR mutations comprised 61.5% of total mutations, and EGFR exon 19 deletion represented ~45% of the total EGFR mutations [3,4]. EGFR is a transmembrane tyrosine kinase receptor that belongs to the ErbB family [5]. EGFR is activated by its ligand, EGF, which leads to receptor dimerization and autophosphorylation on multiple tyrosine residues in the C-terminal tail, and is involved in cell survival and proliferation [6]. Most EGFR mutations and deletions cause ligand-independent dimerization and constitutive activation [7]. Constitutive EGFR signaling is associated with a poor prognosis in NSCLC patients [8]. EGFR tyrosine kinase inhibitors (TKIs), such as gefitinib, were designed to combine ATP binding domains and block EGFR activation [8]. The EGFR exon 19 deletion confers sensitivity to EGFR TKIs, and patients harboring this mutation have higher response rates in clinical trials [8]. However, after EGFR-TKI treatment, most patients develop drug resistance within nine to 14 months [8]. Accumulating studies have reported possible mechanisms for drug resistance, including secondary gene mutations of EGFR (exon 20 T790M mutation), third gene mutations (exon 19 C797S mutation), and wild type/mutant type EGFR amplification [9,10,11].

Two major energy production pathways, oxidative phosphorylation (OXPHOS) and glycolysis, are involved in cell metabolism. Cells utilize both OXPHOS and glycolysis in the presence of oxygen and rely on anaerobic glycolysis under hypoxic conditions. Cells uptake glucose and then convert it to pyruvate. Under limited amounts of oxygen, lactate dehydrogenase A (LDHA) converts pyruvate to lactate, which produces two ATP molecules in the cytosol. Lactate is further secreted into the extracellular space by monocarboxylate transporter 4 (MCT-4). This pathway is called anaerobic glycolysis. In OXPHOS, pyruvate is converted to acetyl-CoA by pyruvate dehydrogenase (PDH) in the mitochondria. Acetyl-CoA then enters the tricarboxylic acid cycle (TCA cycle). NADH and FADH2, produced by the TCA cycle, are oxidized to power ATP production. The metabolism in cancers was studied by Otto Warburg in the 1930s [12]. He found that cancer cells prefer to undergo aerobic glycolysis, which is now known as the Warburg effect, and he assumed that cancer cells prefer aerobic glycolysis to OXPHOS due to a mitochondrial defect [12,13]. Since then, many studies have demonstrated elevated levels of LDHA, MCTs and pyruvate dehydrogenase kinase 1 (PDK1) (which inactivates PDH), which are considered hallmarks of cancer cells [14,15,16].

In recent decades, the Warburg effect has been found to describe the energetic profile of most types of cancer cell. Few studies have shown that alternative metabolism is highly linked to drug-resistant cancer [17]. However, some studies reported that cancer cells did not sacrifice OXPHOS to enhance glycolysis and the rates of oxygen consumption are similar to normal tissue [18,19,20]. Furthermore, studies have shown that some cancer cells prefer to undergo OXPHOS and uptake pyruvate and lactate, which are secreted from adjacent cancer-associated fibroblasts (CAFs), by MCT-1. Lactate is further converted to pyruvate by LDHB and enters the OXPHOS pathway. This is called the reverse Warburg effect [21]. Nevertheless, a study indicated that cancer cells do not exclude OXPHOS or glycolysis, but rather alternatively undergo OXPHOS or glycolysis depending on the presence of normoxia or hypoxia [22]. A clinical study also showed that the metabolic pathway in NSCLC patients was heterogeneous [23]. Furthermore, the authors found that lactate was translocated into the cytosol by MCT-1. Lactate oxidation might fuel the TCA cycle in NSCLC patients [24]. In contrast to studies on the Warburg effect and reverse Warburg effect, some studies have demonstrated a self-sufficient cancer cell metabolism [25,26]. In this case, lactate enters the mitochondria and can be oxidized to pyruvate and then acetyl-CoA by LDHB. Thus, cancer cells have diverse energy production pathways.

A study showed that EGFR translocates to mitochondria in response to stress and TKI treatment in cancer cells [27]. Cancer cells with augmented mitochondrial EGFR exhibited significantly higher resistance to gefitinib than control cells [27]. Moreover, the overexpression of mitochondrial EGFR caused mitochondrial fragmentation [28]. The fusion-deficient mitochondria diminished ATP levels and downregulated OXPHOS [29]. Recently, studies examined the impact of the mitochondrial oxidative metabolism on tumor cell pathophysiology. A remarkable increase in the cellular oxygen consumption rate (OCR) has been demonstrated in several types of cancer, such as lung adenocarcinoma, ovarian cancer and breast cancer. This result suggested that mitochondrial OXPHOS could be a promising therapeutic target [30,31]. However, it remains unclear whether there is a discrepancy in OXPHOS between gefitinib-sensitive and gefitinib-resistant NSCLC. Collectively, we aimed to investigate the correlation between EGFR, mitochondrial metabolism and tumorigenesis and to delineate the differences in energy metabolism between TKI-sensitive cancer cells and TKI-resistant cancer cells.

## 2. Results

### 2.1. Ire Cells Are Gefitinib-Resistant Lung Adenocarcinomas

**Figure 1:**
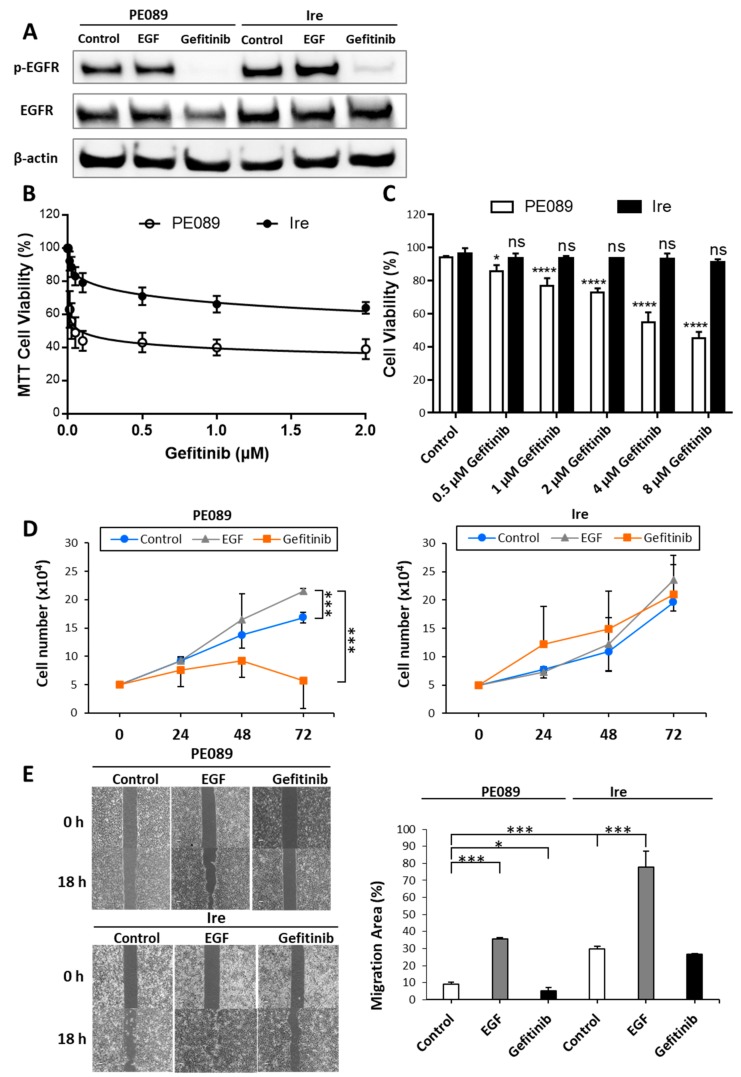
Ire cells are resistant to gefitinib. (**A**) Representative immunoblots of phosphorylated-epidermal growth factor receptor (p-EGFR), EGFR and β-actin are shown. PE089 cells and Ire cells were treated with or without 100 ng/mL EGF and 1 μM gefitinib for 24 h. Decreased p-EGFR levels were observed in gefitinib-treated PE089 cells. (**B**) 3-(4,5-Dimethylthiazol-2-yl)-2,5-diphenyltetrazolium bromide (MTT) assays for PE089 and Ire cells treated with varied concentrations of gefitinib (0 to 2 μM) for 72 h. (**C**) Dose-dependent cell viability assay. Reduction in cell viability was assessed in PE089 cells and Ire cells after different concentrations of gefitinib in 2% Fetal Bovine Serum (FBS) medium for 24 h treatment. The star signs showed significant differences compared to the control group for each cell. (**D**) The cell proliferation of PE089 and Ire cells in the presence of 100 ng/mL EGF or 1 μM gefitinib was examined by trypan blue dye-exclusion method at 0, 24, 48 and 72 h. The control was no EGF or gefitinib treatment. A time-dependent decrease in cell viability was found in the gefitinib-treated PE089 cells, but no response was observed in Ire cells. A significant suppression of cell proliferation was observed in the gefitinib-treated PE089 cells at 72 h. No inhibitory effect on gefitinib-treated Ire cells was observed. (**E**) The effects of 100 ng/mL EGF or 1 μM gefitinib on PE089 and Ire cell motility were measured at 0 and 18 h. The migratory area was calculated and represented as a bar graph. The values represent the mean ± standard deviation (S.D.) from three independent experiments and each experiment performed in duplicate. * *p* < 0.05. *** *p* < 0.001. **** *p* < 0.0001.

To clarify the metabolic difference between nonresistant NSCLC and gefitinib-resistant NSCLC, we used two lung adenocarcinoma cell lines, PE089 cells (gefitinib-sensitive NSCLC) and Ire cells (gefitinib-resistant NSCLC). Resistance to gefitinib was confirmed by immunoblotting (Figure 1A), cell viability (Figure 1B,C), cell proliferation (Figure 1D), and wound healing assays (Figure 1E). PE089 cells and Ire cells were treated with epidermal growth factor (EGF) and gefitinib. After EGF treatment, the protein levels of phosphorylated EGFR (p-EGFR) were increased in both PE089 cells and Ire cells. After gefitinib treatment, the level of p-EGFR was decreased in both PE089 and Ire cells (Figure 1A). However, compared to those in Ire cells, cell viability and cell proliferation were significantly suppressed in PE089 cells after gefitinib treatment (Figure 1B–D). 3-(4,5-Dimethylthiazol-2-yl)-2,5-diphenyltetrazolium bromide (MTT) assay was performed for both PE089 and Ire cells were treated with varying concentrations of gefitinib over a 72 h period. After 72 h treatment with gefitinib, IC50 values for PE089 cells were 85.02 nM (Figure 1B). In addition to the MTT assay, trypan blue assay was also performed to assess cell viability after 24 h gefitinib treatment. Trypan blue assay demonstrated IC50 values of 5.88 μM (Figure 1C) after 24 h gefitinib treatment. For both experiments, concentrations of gefitinib reduced cell viability in PE089 cells, but not in Ire cells (Figure 1B,C). Similarly, concentrations of gefitinib inhibited cell proliferation in PE089 cells, but not in Ire cells (Figure 1D). In the migration assay, the migratory area was increased after EGF treatment in both cells. However, gefitinib significantly inhibited cell motility in only PE089 cells (Figure 1E), and had no significant effect on Ire cells. Overall, the protein level of p-EGFR was reduced in Ire cells; however, cell proliferation, cell viability, and cell motility were shown to have no significant inhibition after gefitinib treatment. Based on the results, we suggested that Ire cells are resistant to gefitinib.

### 2.2. Enhanced Mitochondrial Translocation of EGFR and Mitochondrial Bioenergetics in TKI-Resistant Ire Cells

**Figure 2:**
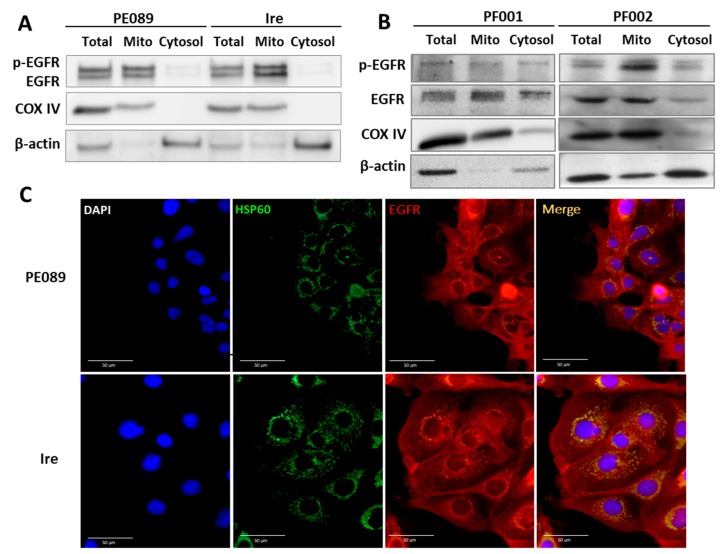
Mitochondrial translocation of EGFR was found in PE089 cells, Ire cells, and lung adenocarcinoma cells. (**A**) The mitochondrial fraction (Mito) and cytosolic fraction (Cytosol) of PE089 and Ire cells were isolated by differential centrifugation. Representative immunoblottings of p-EGFR, EGFR, cytochrome c oxidase subunit IV (COX IV) and β-actin of PE089 and Ire cells are shown. COX IV was used as the mitochondrial marker protein. β-Actin was used as the cytosolic marker protein. Total protein lysate. (**B**) The mitochondrial fraction and cytosolic fraction of the patient-derived PF001 and PF002 cells were purified. PF001 and PF002 cells were collected from patients with EGFR-positive lung adenocarcinoma. (**C**) PE089 cells and Ire cells were immunodetected by anti-EGFR-CF594 (red signals) and anti-HSP60-CF488A (green signals). Nuclei were stained with DAPI (blue signals) (scale bars, 50 μm). The increased mitochondrial mass and the mitochondria-localized EGFR are shown.

Many studies have reported that EGFR can translocate to the cytoplasm [32], mitochondria [27,28,33,34], and the nucleus [35]. One of studies showed that gefitinib can increase the mitochondrial EGFR (mtEGFR) levels in breast cancer cells. Authors also found that breast cancer cells with increased mtEGFR showed more resistance to gefitinib. Thus, we wondered whether levels of mtEGFR were enhanced in gefitinib-resistant Ire cells. To investigate whether mitochondrial translocation of EGFR was present in PE089 cells and Ire cells, we examined the localization of EGFR by subcellular fractionation and immunoblotting. The purity controls for the mitochondrial fraction and cytosol fraction were COX IV and β-actin, respectively. The results demonstrated that both p-EGFR and EGFR were located in the mitochondria in PE089 cells and Ire cells (Figure 2A). In addition, higher protein levels of p-EGFR and EGFR were seen in Ire cells. This result was further validated by immunofluorescent staining (Figure 2C). Mitochondrial EGFR is shown in yellow in fluorescent images merged with green (EGFR) and red fluorescent signals (mitochondrial HSP60). It is worth mentioning that we also found an increased mitochondrial mass and EGFR-positive mitochondria in Ire cells (Figure 2C). Furthermore, we detected mitochondria-accumulated EGFR in patient-derived EGFR-positive lung adenocarcinoma cells (PF001 and PF002) (Figure 2B). The same result showed that PF002, in gefitinib-resistant cells, has increased mtEGFR compared to gefitinib-sensitive PF001.

**Figure 3:**
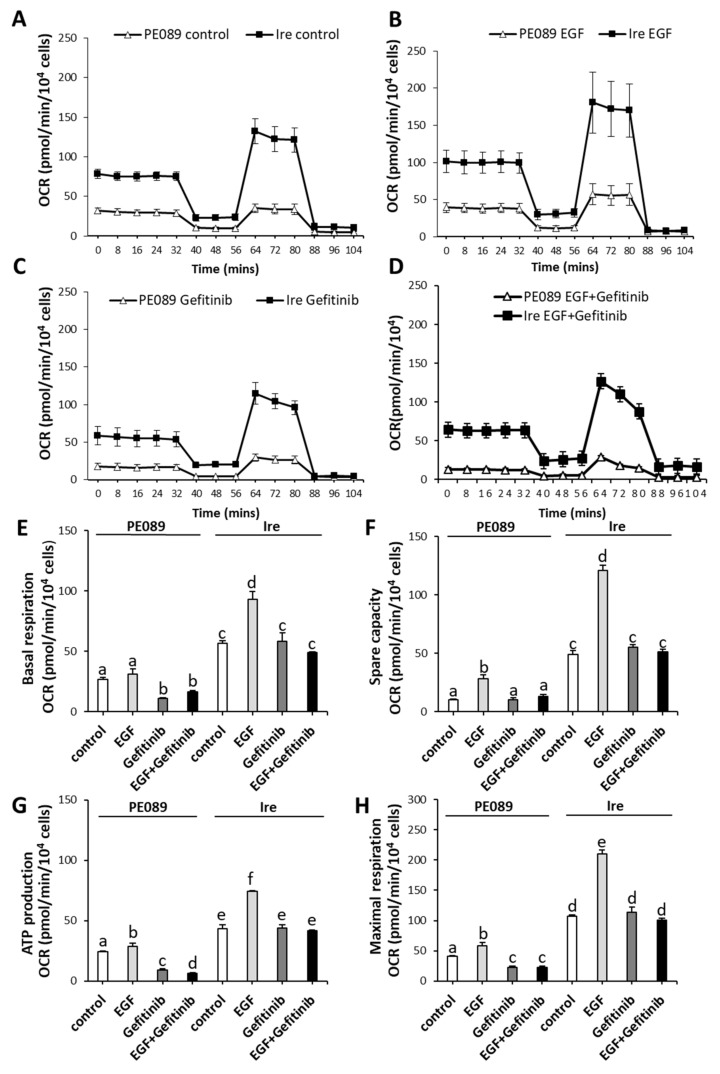
Ire cells exhibited higher mitochondrial bioenergetics. PE089 and Ire cells were treated with or without 100 ng/mL EGF and 1 μM gefitinib for 24 h. The time-course progress curves of the oxygen consumption rate (OCR) are shown in (**A**) the control cells, (**B**) the EGF-treated cells, (**C**) gefitinib-treated cells, and (**D**) combined treatment with EGF and gefitinib. Individual parameters for basal respiration (**E**), spare capacity (**F**), ATP production (**G**), and maximal respiration (**H**) are represented. The values represent the mean ± S.D. from three independent experiments and each experiment performed in duplicate. The presence of the same letters on the bars indicates no significant difference (*p* > 0.05) between the two different conditions and the presence of different letters on the bars indicates a significant difference (*p* < 0.05) between the two different conditions.

Next, we compared the differences in mitochondrial bioenergetics between PE089 cells and Ire cells. We determined the OXPHOS efficiency by measuring mitochondrial respiration using a Seahorse XF24 analyzer (Figure 3). Supplementary Figure S1 illustrates the experiment of mitochondrial bioenergetics by Seahorse XF24. We compared the OCR between PE089 cells and Ire cells in control group (Figure 3A), EGF treatment (Figure 3B), gefitinib treatment (Figure 3C), and combined treatment with EGF and gefitinib (Figure 3D). Ire cells clearly showed a significantly increased OCR of basal respiration (2.10-fold), spare capacity (4.73-fold), ATP production (1.77-fold) and maximal respiration (2.64-fold) compared to PE089 cells (Figure 3E–H). In Ire cells, EGF treatment increased basal respiration (1.64-fold), spare capacity (2.48-fold), ATP production (1.71-fold) and maximal respiration (1.96-fold) compared to those in the Ire control group. However, EGF treatment only increased spare capacity (2.71-fold) and maximal respiration (1.44-fold) in PE089 cells when compared to the PE089 control group. Gefitinib treatment significantly reduced the OCR of basal respiration (2.40-fold), ATP production (2.60-fold) and maximal respiration (1.76-fold) in PE089 cells, but there was no significant inhibition of mitochondrial bioenergetics in Ire cells. Combined treatment with EGF and gefitinib caused significantly decreased ATP production in PE089 cells but there were no inhibitory effects in Ire cells (Figure 3G). Thus, we propose that no inhibition by gefitinib of mitochondria-translocated p-EGFR and EGFR might concurrently contribute to mitochondrial OXPHOS efficiency in Ire cells.

### 2.3. Higher Activity and Higher mRNA and Protein Expression Levels in Mitochondrial Complexes in TKI-Resistant Ire Cells

To understand the activity of which mitochondrial respiratory complex leads to higher mitochondrial bioenergetics in Ire cells than PE089 cells, we examined the individual activities of mitochondrial respiratory complexes I-V. We measured mitochondrial complex activity through Seahorse XF24 by differentially adding substrates and inhibitors for specific respiratory complexes (Supplementary Figure S1).

**Figure 4:**
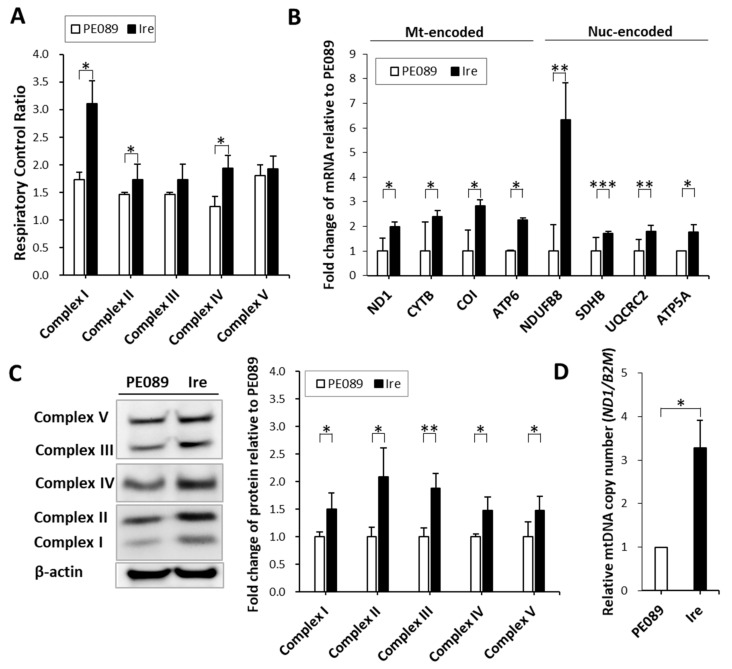
Representative changes in the respiratory activity and the transcripts and protein levels of mitochondrial respiratory complexes. (**A**) Mitochondrial complexes activity was analyzed by Seahorse XF24 analyzer. (**B**) The differential changes in the transcripts of mitochondria-encoded respiratory complex genes (*ND1*, *CYTB*, *COI* and *ATP6*) and nucleus-encoded respiratory complex genes (*NDUFB8*, *SDHB*, *UQCRC2* and *ATP5A*) were measured by qPCR. The expression levels were normalized to an internal control (*ACTB*) and fold changes were relative to the control. (**C**) Immunoblots of mitochondrial respiratory complex proteins are shown. Antibody cocktails (MS604) against mitochondrial complex subunits were used to examine the expression profiles of mitochondrial respiratory complexes. The five distinct bands in the immunoblots represent Complex I subunit NDUFB8 (~20 kD, nuclear encoded), Complex II subunit (~30 kD, nuclear encoded), Complex III subunit Core 2 (~47 kD, nuclear encoded), Complex IV subunit I (~39 kD, mitochondrial encoded), and ATP synthase subunit alpha (~53 kD, nuclear encoded). All the proteins were increased in the mitochondrial-encoded or nuclear-encoded mitochondrial respiratory complex proteins. β-Actin was used as a loading control. (**D**) Fold change in mitochondrial DNA (mtDNA) copy number relative to that of PE089 cells. *ND1* was used to represent the mtDNA copy number. *B2M* was used as an internal control gene of nuclear DNA. The values represent the mean ± S.D. from three independent experiments and each experiment performed in duplicate. * *p* < 0.05. ** *p* < 0.01. *** *p* < 0.001.

In Figure 4A, the data showed that the activities of complexes I to V were enhanced 1.79-fold, 1.18-fold, 1.18-fold, 1.57-fold and 1.06-fold, respectively, in Ire cells. Furthermore, we measured changes in the mRNA levels of respiratory complex subunits by qPCR. Higher transcript levels were found in Ire cells regardless of which mitochondria-encoded complex genes (*ND1*, *CYTB*, *COI* and *ATP6*) and nucleus-encoded genes (*NDUFB8*, *SDHB*, *UQCRC2* and *ATP5A*) were measured (Figure 4B). We further investigated the protein levels of mitochondrial complex subunits using immunoblotting. We used a human OXPHOS antibody cocktail to detect NDUFB8 (nucleus-encoded complex I subunit), SDHB (nucleus-encoded complex II subunit), UQCRC2 (nucleus-encoded complex III subunit), COX II (mitochondria-encoded complex IV subunit) and ATP5A (nucleus-encoded complex V subunit). The protein levels of complex I to complex V were significantly increased by 1.50-, 2.08-, 1.88-, 1.47- and 1.47-fold in Ire cells, respectively (Figure 4C). In addition, the mitochondrial DNA (mtDNA) and copy number (ND1 gene) was also enhanced in Ire cells (Figure 4D). All these results represented the higher activities and enhanced expression of mitochondrial respiratory complexes in Ire cells that harbored a higher mtEGFR.

### 2.4. Gefitinib-Resistant Ire Cells Relied on the OXPHOS Pathway to Generate ATP

**Figure 5:**
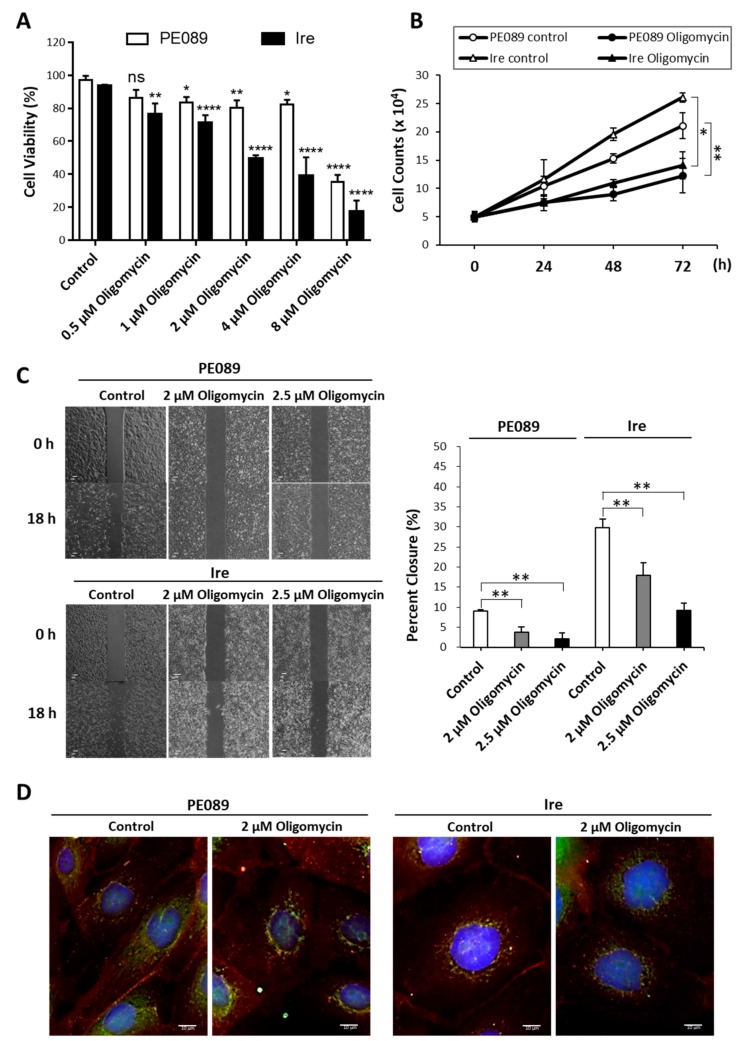
Mitochondrial bioenergetics contributed to the cell survival, cell proliferation, and cell motility of PE089 and Ire cells. (**A**) Dose-dependent cell viability assay. Reduction in cell viability was assessed in PE089 cells and Ire cells after different concentrations of oligomycin in 2% FBS medium. The star signs showed the significant differences compared to the control group for each cell. (**B**) Proliferation of PE089 and Ire cells in response to 2 μM oligomycin for 0, 24, 48, and 72 h was measured by the trypan blue-dye exclusion method. (**C**) The cell motility of PE089 and Ire cells in response to 2 or 2.5 μM oligomycin was determined by wound healing assay. Images were taken at 0 and 18 h, and the bar chart was calculated from the images. Significant inhibition by oligomycin of cell proliferation, cell survival, and cell motility was found in both PE089 and Ire cells. (**D**) PE089 and Ire cells were subjected to immunofluorescent staining with or without 2 μM oligomycin for 24 h. The cells were immunodetected by anti-EGFR-CF594 (red signals) and anti-HSP60-CF488A (green signals). Cell nuclei were stained with DAPI (blue signals) (scale bars, 10 μm). The control is no oligomycin treatment. The values represent the mean ± S.D. from three independent experiments and each experiment performed in duplicate. * *p* < 0.05. ** *p* < 0.01. **** *p* < 0.0001.

To determine whether gefitinib-resistant Ire cells rely more on OXPHOS than glycolysis for cell metabolism, we determined the effects of the ATP synthase inhibitor oligomycin on cell proliferation, cell viability, and cell motility. A dose-dependent treatment decreased in cell viability in 2% FBS was observed in Ire cells and PE089 cells (Figure 5A). The IC_50_ of inhibitions are measured at an oligomycin concentration of 6.935 μM in PE089 cells and 2.099 μM in Ire cells. A time-dependent response, with 2 μM oligomycin and reduced cell proliferation, were shown in PE089 cells and Ire cells (Figure 5B). Moreover, oligomycin significantly reduced cell migratory ability in both cell types (Figure 5C). In addition, we found that most fragmented mitochondria were found in the oligomycin-treated cells (Figure 5D). These results demonstrated that Ire cells may rely more on OXPHOS than glycolysis to generate ATP for energy-demanding processes.

### 2.5. Enhanced Expression of OXPHOS Pathway-Related Genes in Ire Cells

**Figure 6:**
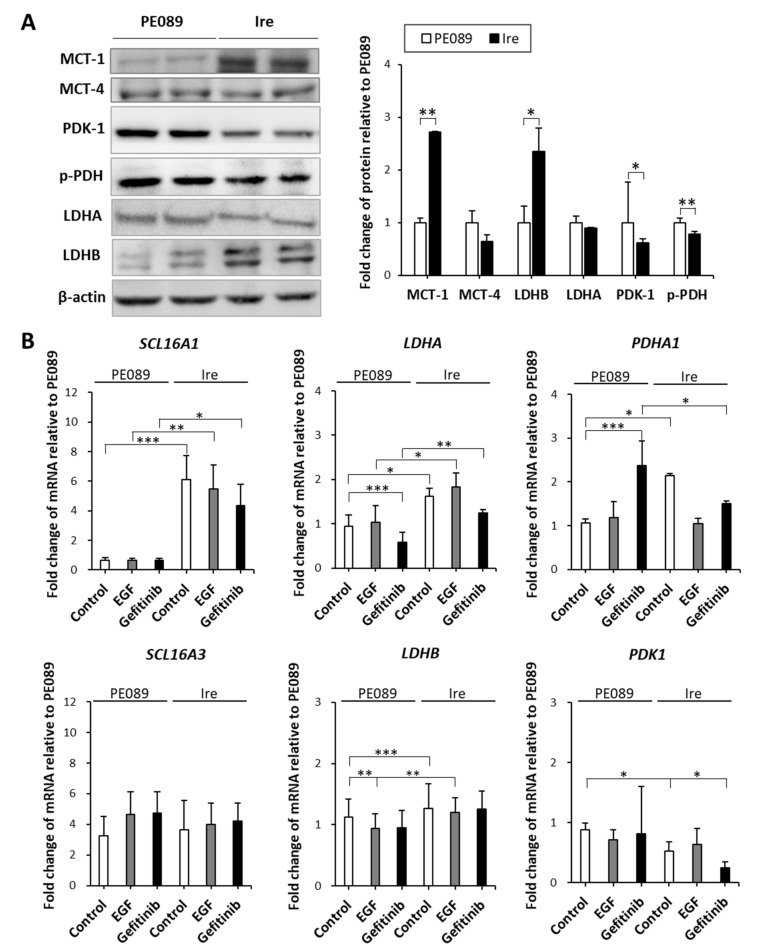
Differential expression levels of OXPHOS-related regulators in PE089 and Ire cells. (**A**) Representative immunoblots for MCT-1, MCT-4, PDK-1, p-PDH E1α, LDHA and LDHB are shown. β-Actin was used as a loading control. Significantly higher levels of MCT-1 and LDHB and decreased levels of PDK-1 and p-PDH were found in Ire cells. (**B**) The differential expression levels of *SCL16A1* (encodes MCT-1), *SCL16A3* (encodes MCT-4), *LDHA*, *LDHB*, *PDHA1* and *PDK1* mRNA were determined by real-time qPCR. The expression levels were normalized to an internal control (*ACTB*) and fold changes were relative to the control. The values represent the mean ± S.D. from three independent experiments and each experiment performed in duplicate. * *p* < 0.05. ** *p* < 0.01. *** *p* < 0.001.

Conventionally, researchers have shown that cancer cells tend to generate ATP via the aerobic glycolytic pathway. Recently, researchers have demonstrated heterogeneity in the lung cancer metabolism [23]. As mentioned above, our results showed that Ire cells relied more on OXPHOS than glycolysis to generate ATP. To understand whether OXPHOS pathway-related genes were coordinately regulated in Ire cells, we investigated the expression of OXPHOS-related proteins MCT-1, MCT-4, PDK-1, p-PDH E1α, LDHA, and LDHB, through immunoblotting. In Figure 6A, the protein expression levels of MCT-1 (2.71-fold) and LDHB (2.35-fold) were significantly increased in Ire cells. Lower protein expression levels of PDK-1 (1.61-fold) and p-PDH E1α (1.28-fold) were found in Ire cells. However, the protein expression of MCT-4 and LDHA did not differ between PE089 and Ire cells. We also examined gene expression by qPCR (Figure 6B). The expression levels of the OXPHOS-related genes *SCL16A1* (which encodes MCT-1), *LDHA*, *LDHB* and *PDHA1* were increased by 6.09-, 1.61-, 1.26- and 2.14-fold in Ire cells, respectively. However, a 1.67-fold decrease in PDK1 was found in Ire cells, and no significant difference in *SCL16A3* (which encodes MCT-4) was found between PE089 cells and Ire cells. These results indicate that Ire cells preferred the MCT-1- and LDHB-related pathways to the MCT-4- and LDHA-related pathways.

### 2.6. MCT-1 Can Serve as a Therapeutic Target for Gefitinib-Resistant Ire Cells In Vitro

**Figure 7:**
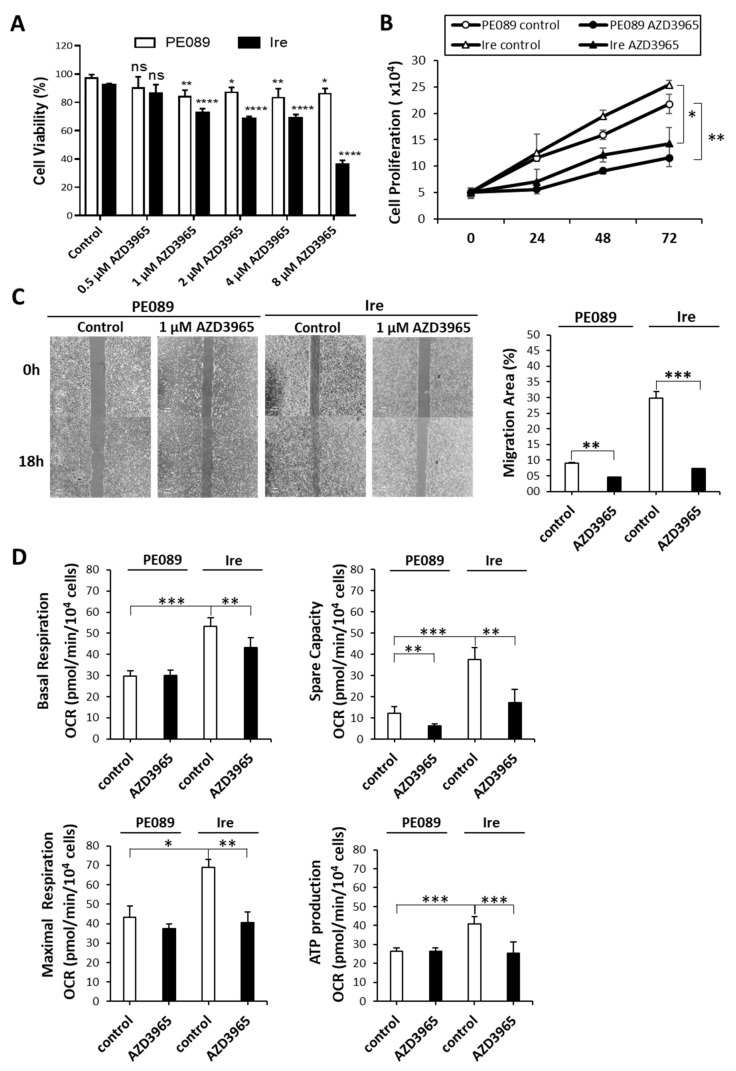
AZD3965 exhibited inhibitory effects on the cell growth and mitochondrial bioenergetics of Ire cells. (**A**) Dose-dependent cell viability assay. Reduction in cell viability was assessed in PE089 cells and Ire cells after different concentrations of AZD3965 in 2% FBS medium. The star signs showed significant differences compared to a control group for each cell. (**B**) The proliferation of PE089 and Ire cells in response to 1 μM AZD3965 for 0, 24, 48 and 72 h was measured by the trypan blue-dye exclusion method. (**C**) The migratory ability of PE089 and Ire cells in response to 1 μM AZD3965 was determined by a migration assay. Cell images were taken at 0 and 18 h, and the migration area was measured by ImageJ. Significant inhibition by AZD3965 of cell proliferation, cell survival, and cell motility were observed in both PE089 and Ire cells. (**D**) The effects of AZD3965 on the mitochondrial bioenergetics of PE089 and Ire cells were examined. Individual parameters of OCR for basal respiration, spare capacity, ATP production and maximal respiration were analyzed by XF24 extracellular flux analyzer. The values represent the mean ± S.D. from three independent experiments and each experiment performed in duplicate. * *p* < 0.05. ** *p* < 0.01. *** *p* < 0.001.

MCT-1 has been demonstrated to be a potential cancer biomarker, offering a therapeutic target for cancer treatment [36]. Thus, we treated cells with AZD3965, which is an MCT-1 inhibitor currently in clinical trials. A dose-dependent treatment decreasing cell viability in 2% FBS was observed in Ire cells but not in PE089 cells (Figure 7A). We used 1 μM AZD3965 for further experiment. The IC_50_ of inhibition is measured at an AZD3965 concentration of 5.747 μM in Ire cells. Moreover, in the time-dependent proliferation assay, AZD3965 treatment significantly reduced cell growth in both cell lines, especially in gefitinib-resistant Ire cells (Figure 7B). In the cell migration assay, AZD3965 treatment also decreased the migratory ability of both cells (Figure 7C). We further investigated the effects of AZD3965 on mitochondrial bioenergetics. AZD3965 treatment significantly reduced the OCR of basal respiration, spare capacity, maximal respiration and ATP production in Ire cells (Figure 7D). However, AZD3965 treatment only reduced spare capacity in PE089 cells (Figure 7D). Collectively, these results support the possibility that AZD3965 treatment may be an efficient therapy to overcome gefitinib-resistant NSCLC.

## 3. Discussion

In NSCLC, EGFR mutations caused ligand-independent activation of EGFR [7]. Activation of EGFR results in the activation of multiple downstream pathways, which induce cell survival and cell proliferation [37]. This phenomenon is associated with poor prognosis in NSCLC [8]. Gefitinib is an EGFR tyrosine kinase inhibitor that has been approved for the treatment of NSCLC patients with EGFR mutations [8]. Despite the remarkable effect of this compound, NSCLC patients often develop resistance to gefitinib [8]. Although second- or third-generation EGFR-TKIs have been developed, drug resistance remains a major obstacle for NSCLC treatment. Many studies have delineated the mechanisms of drug-resistant NSCLC; however, the molecular metabolism of drug-resistant NSCLC is still unclear. In the present study, we found that Ire cells prefer to undergo oxidative phosphorylation rather than glycolysis for ATP generation. The preference for oxidative phosphorylation can be illustrated by the oligomycin-mediated obstruction of the electron transport chain, leading to suppressed cancer cell proliferation and cell survival. It is noteworthy that the increase in MCT-1 was greater than MCT-4 in Ire cells (Figure 6B). Treatment with the MCT-1 inhibitor AZD3965 further reduced cell proliferation, presumably by blocking the compensatory switch to oxidative phosphorylation metabolism in PE089 and Ire cells (Figure 7). Taken together, these results demonstrated that targeting MCT-1 might be a potential strategy for NSCLC.

EGFR is a membrane protein, and numerous studies have revealed that it can translocate to the cytoplasm [32] and mitochondria [27,28,33,34], and can be shuttled to the nucleus [35,38] in cancers. In the present study, we observed nuclear EGFR and mitochondrial EGFR in both PE089 cells and Ire cells (Figure 2A,C). Previous studies showed that nuclear EGFR happened in cetaximub-resistant lung cancer and in gefitinib-resistant breast cancer [35,38]. Here, we focused on the mitochondrial translocation of EGFR. Some studies have reported that EGFR can translocate to mitochondria after EGF treatment [28,34]. EGF-induced mtEGFR decreased cytochrome *c* oxidase subunit II (COX II) activity by the phosphorylation of COX II and decreased cellular ATP in murine fibroblasts [33,34]. However, another study showed that the enhanced expression of mitochondria-targeting EGFR upregulated cellular ATP and cell motility in NSCLC. Moreover, enhanced mtEGFR caused mitochondrial fragmentation and the redistribution of lamellipodia for NSCLC cell migration, which might contribute to cancer metastasis and poor overall survival in vivo [28]. In breast cancer, studies have demonstrated that gefitinib treatment induces EGFR translocation to the mitochondria. It is noteworthy that breast cancer cells with a higher mtEGFR are significantly more resistant to gefitinib [27]. In contrast, we found ligand-independent mitochondrial translocation of EGFR in both gefitinib-sensitive and gefitinib-resistant NSCLC cells. In fact, we noticed that the level of mtEGFR was higher in the gefitinib-resistant Ire cells than in parental PE089 cells (Figure 2). Consistent with these findings, we found increased cell proliferation, motility, and mitochondrial fragmentation in Ire cells with increased mtEGFR without EGF stimulation (Figure 1D,E and Figure 2A). We further assessed the mitochondrial metabolism of both cell lines. The results demonstrated that gefitinib-resistant Ire cells had a higher mitochondrial respiratory capacity compared to parental PE089 cells (Figure 3). Moreover, EGF treatment enhanced the mitochondrial OCR in both PE089 cells and Ire cells. Among them, ATP production was significantly increased after EGF treatment (Figure 3G). A recent study showed that gefitinib treatment enhanced the mitochondrial membrane potential (MMP) and mitochondrial dehydrogenase activity of gefitinib-resistant NSCLC, but those were decreased in gefitinib-sensitive NSCLC [39]. In contrast, in the present study, gefitinib downregulated the mitochondrial function in gefitinib-sensitive PE089 cells. However, it did not upregulate mitochondrial respiration or ATP content in gefitinib-resistant Ire cells. Here, the lack of phosphorylated EGFR upon gefitinib treatment was not consistent with the unaltered mitochondrial metabolism in gefitinib-resistant Ire cells.

In addition to mitochondrial bioenergy, we also determined the activities of mitochondrial respiratory complexes in both gefitinib-sensitive NSCLC and gefitinib-resistant NSCLC cells (Figure 4A). Higher activities of respiratory complexes I, III, and IV were found in Ire cells than in PE089 cells. Notably, both the activity and expression level of respiratory complex I were significantly increased in Ire cells (Figure 4). Numerous studies have attempted to understand the role of respiratory complex I in lung cancers [40,41,42,43,44]. It was demonstrated that 45% of lung cancers tested harbored mitochondrial DNA mutations affecting respiratory complexes, of which approximately 57% were in respiratory complex I, 18% in respiratory complex III, and 25% in respiratory complex IV [40]. Among them, respiratory complex I mutations accounted for the highest proportion of mitochondrial complex mutations. Furthermore, a study showed that mtDNA mutations of complex I subunits represent deficient respiratory activity, induce ROS generation, enhance cell metastasis, and increase resistance to apoptosis in various types of cancer [41]. Another study also described that the inhibition of complex I by knockdown or rotenone treatment enhanced cancer metastasis, tumor growth, and ROS generation in HeLa cells [42]. However, others have reported that the inhibition of respiratory complex I suppressed cancer metastasis and cancer growth in NSCLC [43,44]. A study revealed that cisplatin-resistant cells have increased complex I activity, MMP, and cellular ATP, implying that the activation of mitochondria leads to enhanced cell metastasis compared to parental cells [43]. In addition to complex I, the OXPHOS activity and expression levels of other respiratory complexes were upregulated in Ire cells (Figure 4A,B). This finding was consistent with previous studies showing that the enhanced expression of OXPHOS genes is negatively correlated with the prognosis, which is correlated with cell metastasis, of lung adenocarcinoma [43,45]. Therefore, we concluded that respiratory complex I plays an important role in NSCLC.

Traditionally, researchers assumed that cancer cells prefer to generate ATP through aerobic glycolysis because of a mitochondrial defect [12,13]. However, an increasing number of studies have suggested that cancer cells do not sacrifice OXPHOS to enhance glycolysis [18,19,20]. The OCR of cancer cells is similar to that of normal tissues [19,20]. Cancer cells can alter their reliance on OXPHOS or glycolysis depending on normoxia or hypoxia [22]. Furthermore, a drug-inhibiting mitochondria can be a potential therapy for cancers with high rates of OXPHOS [43,46]. Our data showed that the gefitinib-resistant NSCLC cells Ire had higher mitochondrial OXPHOS (Figure 3 and Figure 4). We postulated that gefitinib-resistant Ire cells preferred to generate ATP through OXPHOS rather than glycolysis. To confirm this hypothesis, we treated cells with the ATP synthase inhibitor oligomycin to investigate the pathway for ATP synthesis. Previously, a study revealed that oligomycin treatment completely suppressed OXPHOS and shifted the metabolism from OXPHOS to glycolysis in NSCLC with a high rate of respiration [47]. The results showed that oligomycin inhibited cell proliferation but did not arrest cell division and survival in long-term treatment. However, in our study, oligomycin treatment significantly suppressed not only cell proliferation but also cell viability and cell motility in Ire cells (Figure 5A–C). In addition, oligomycin treatment induced mitochondrial morphology into the fission form (Figure 5D). Overall, we suggest that ATP generated from OXPHOS is essential for gefitinib-resistant NSCLC.

Aerobic glycolysis is regulated by several oncogenic signaling pathways. Studies have reported that HIF1-induced LDHA and PDK-1 cause the Warburg effect, which enhances glucose metabolism in cancer cells [14,48]. Elevated levels of LDHA, PDK-1, and MCT-4 are considered hallmarks of cancers with Warburg Effect [14,15,16]. Tumor cells generally export the excessive lactate along with protons via MCT-4 instead of utilizing it as a nutrient. In contrast, another study reported that LDHB and MCT-1 are also involved in cancer metabolism. In HeLa cells, lactate can be reversed to pyruvate by LDHB, and part of LDHB was localized in the mitochondria [26]. The study showed that the activity of LDHB in mitochondrial lysate was higher than in whole-cell lysate. In addition, it has been shown that breast cancers uptake lactate, which is secreted through MCT-1 from adjacent fibroblast cells [21]. In NSCLC, a study demonstrated that cancer cells uptake exogenous lactate via MCT-1 without fibroblast cell coculture [24]. In the present study, we determined the expression changes in these metabolic proteins (Figure 6). The results showed that the expression levels of PDK-1 and p-PDH were decreased in Ire cells. It is noteworthy that MCT-1 and LDHB were significantly increased in Ire cells. A previous study elucidated that MCT-1 levels are enhanced in malignant lung cancer cells compared to adjacent nonmalignant lung tissue, particularly in later stages [49]. Moreover, the presence of MCT-1-positive tumors was highly correlated with low overall patient survival [50]. Therefore, inhibition of MCT-1 could be considered as a potential targeted therapy in cancers. AZD3965 is an MCT-1-specific inhibitor and has been used in clinical trials [51]. AZD3965 was developed for patients with solid tumors or lymphomas, such as diffuse large B-cell lymphoma and Burkitt’s lymphoma [51,52]. A study showed that Burkitt’s lymphoma, mostly MCT-1 positive and MCT-4 negative, was strongly inhibited by AZD3965 in vivo [52]. In addition, the authors found that AZD3965 treatment caused an increase in OXPHOS and the TCA cycle in vitro [52]. Another study showed that some SCLC cells responded to AZD3965, particularly those with a high MCT-1 but a lack of MCT-4 [51]. The authors indicated that MCT-4 overexpression caused resistance to AZD3965. Thus, cancer cells with MCT-1 expression but an absence of MCT-4 were predicted to be an AZD3965-sensitive population [51]. In our study, the expression of MCT-1 was 2.71 times higher in Ire cells than in PE089 cells (Figure 6A). However, the expression of MCT-4 did not differ between PE089 cells and Ire cells (Figure 6A). We treated both cells with AZD3965. AZD3965 treatment decreased the proliferation, viability, and motility of both cell lines (Figure 7A–C). In contrast, OXPHOS declined basal respiration, spare capacity, ATP production, and maximal respiration in Ire cells but only declined spare capacity in PE089 cells after AZD3965 treatment (Figure 7D).

Understanding the molecular mechanisms of the differential phenotypes during the development of TKI resistance can further help the development of therapeutic strategies. In the present study, we found that metabolic reprogramming occurs during the development of TKI-resistant NSCLC. A preference for the glucose metabolism shift from glycolysis to oxidative phosphorylation was discovered in the parental gefitinib-sensitive PE089 cells and PE089-derived gefitinib-resistant Ire cells. Overall, we found that gefitinib-resistant NSCLC is characterized by a ligand-independent EGFR translocation to mitochondria, which might contribute to the upregulation of mitochondrial function and OXPHOS capacity. Concomitantly, the levels of OXPHOS-related proteins, especially MCT-1, were increased in Ire cells. AZD3965 can be a therapeutic target for NSCLC and can be used in clinical trials.

## 4. Materials and Methods

### 4.1. Chemicals and Reagents

Most chemicals were purchased from Sigma-Aldrich (St. Louis, MO, USA) including ADP (A5285), antimycin A (A8647), duroquinone (D223204), L-ascorbic acid (A5960), malic acid (M1000), *N,N,N*′,*N*′-tetramethyl-p-phenylenediamine (TMPD) (T7394), oligomycin (O4876), rotenone (R8875), sodium azide (S2002), sodium pyruvate (P5280) and succinic acid. AZD3965 (1448671-31-5) was purchased from Medchem Express (Monmouth Junction, NJ, USA). Gefitinib (184475-35-2) was purchased from Invivogen (San Diego, CA, USA). Recombinant perfringolysin O, a plasma membrane permeabilizer (PMP), was purchased from Agilent Technology (Santa Clara, CA, USA). The antibodies and the sources are listed in Table 1. We prepared the gefitinib stock solution by dissolving in DMSO (50 mM). The final working solution is 1 μM in 0.002% DMSO. In order to confirm a safe concentration of DMSO, we performed the cell viability assay with 0.005%, 0.01%, and 0.02% DMSO. The cell viability results were surpassed by >90% when the concentration of DMSO was less than 0.02% (Supplementary Figure S1).

### 4.2. Cell Culture

The PE089 cell line was derived from the pleural effusion of a female patient with lung adenocarcinoma. PE089 cells harbor *EGFR* exon 19 deletion and are sensitive to the first-generation EGFR-TKI, gefitinib. PE089 cells were cultured with 1 μM gefitinib for three months to produce the resistant cell lines, which were named Ire cells. Ire cells were stable in the absence of gefitinib during subsequent maintenance. Ire cells harbor *EGFR* exon 19 deletion but no T790M mutation and are resistant to gefitinib. Both cell lines were obtained from Dr. K. J. Liu (National Institute of Cancer Research, National Health Research Institutes, Taiwan). PE089 and Ire cells were maintained in Minimum Essential Medium (MEM, Thermo Fisher Scientific, Waltham, MA, USA) with 10% FBS (Thermo Fisher Scientific), 1X GlutaMax (Thermo Fisher Scientific) and 1X penicillin/streptomycin (PS, Thermo Fisher Scientific). PF001 and PF002 were the cells collected from NSCLC patients with malignant pleural effusions. The PF001 cells are gefitinib sensitive. They were collected from a 69-year-old male patient with lung adenocarcinoma harboring EGFR Exon 21: L858R mutation, who had the malignant pleural effusion as an initial manifestation. The PE002 cells are gefitinib resistant. They were collected from a 48-year-old male patient with lung adenocarcinoma also harboring EGFR Exon 21: L858R mutation, with the malignant pleural effusion as recurrence. This prospective study was approved by the Institutional Review Board of the Sun Yat-Sen Cancer Center and by the hospital’s Ethics Committee. It was conducted in accordance with the ethical principles of the Declaration of Helsinki and guidelines on good clinical practice. All of the patients provided written informed consent.

### 4.3. Immunoblotting

Cells were lysed in lysis buffer (150 mM NaCl, 1 mM EDTA pH 8.0, 50 mM Tris-HCl pH 8.0, 1% NP-40, 0.5% sodium deoxycholate (DOS), 0.1% sodium dodecyl sulfate (SDS), 0.1 mM phenylmethylsulfonyl fluoride (PMSF), 1 μg/mL leupeptin, and 1 μg/mL aprotinin). Protein was quantified by Bradford method (Bio-Rad, Hercules, CA, USA). Protein sample (40 μg) was loaded and separated by SDS-polyacrylamide gel electrophoresis (PAGE). The primary antibodies used for immunohistochemistry are listed in Table 1. Secondary antibodies were applied followed by enhanced chemiluminescence detection (ECL) using an ECL system (GE Healthcare Life Sciences, Chicago, IL, USA). Images were captured with ImageQuant LAS4000 (GE Healthcare Life Sciences). Densitometric measurements of immunoblotting bands were performed with the ImageJ program.

### 4.4. Cell Proliferation

The proliferation of PE089 and Ire cells was quantified by trypan blue staining. Aliquots of 5 × 10^4^ PE089 cells and 3 × 10^4^ Ire cells were suspended in culture medium and seeded in 24-well culture plates. After incubation at 37 °C for 0, 24, 48, and 72 h, cells were collected and mixed in the ratio 1:1 with 0.4% trypan blue buffer (Sigma-Aldrich). A total of 10 μL of the cell mixture was placed in a hemocytometer, and the cell numbers were counted under microscopy.

### 4.5. Cell Viability

The viability of cells was determined by trypan blue. Aliquots of 5 × 10^4^ PE089 cells and 3 × 10^4^ Ire cells were suspended in cultured medium and seeded in 24-well culture plates. After cell attachment, cells were treated with drugs for 0, 24, 48, and 72 h. Then, cells were harvested and mixed with 0.4% trypan blue buffer (Sigma-Aldrich). The number of cells was counted under microscopy. The IC_50_ cell viability was analyzed by MTT assay. Cells were seeded in 96-well plates and 24 h later gefitinib were added at different concentrations (0–2 μM) for an additional 72 h. MTT metabolite was performed at OD 540 nm and mean ± standard deviation values were plotted on a graph.

### 4.6. Wound Healing Assay

The wound healing assay was set up with an Ibidi Culture-Insert (Ibidi GmbH, Gräfelfing, Germany). First, inserts were placed on the bottom of the dish. PE089 or Ire cells were seeded in the inserts and maintained for 24 h to allow cell attachment. After cell attachment, the inserts were removed, and the cells were washed with 1× PBS. Then, the cells were incubated in serum-free medium with drugs for 18 h and observed under microscopy. Wound areas were quantified by ImageJ.

### 4.7. Mitochondrial Extraction

A mitochondrial isolation buffer (MI buffer) was prepared with 25 mM HEPES KOH (pH 7.5), 5 mM MgCl_2_, 0.5 mM EDTA, and 75 mM sucrose in diH_2_O. Prior to extraction, 1 M DTT, 1 mg/mL leupeptin, 1 mg/mL aprotinin and 100 mM PMSF were added to the MI buffer. The mitochondrial fraction was extracted with the MI buffer using a 26 G syringe to break the cell membrane. The supernatant was collected after serial differential centrifugation at 1000× *g* for 10 min at 4 °C twice and transferred to a new tube on ice for further centrifugation at 8000× *g* for 10 min at 4 °C. The pellets were collected, resuspended in MI buffer, and centrifuged at 8000× *g* for 10 min at 4 °C. The mitochondrial fraction was enriched in the final pellet.

### 4.8. Immunofluorescence Analysis

Cells were grown on glass coverslips, fixed with 4% paraformaldehyde and permeabilized with 0.2% Triton X-100. Cells were cultured with 5% BSA to block nonspecific binding of the antibodies. The antibodies used in immunofluorescence are listed in Table 1. Then, the cells were incubated overnight with primary antibodies anti-EGFR and anti-HSP60 at a dilution of 1:200 at 4 °C. Secondary antibodies anti-mouse IgG-CF488 and anti-rabbit IgG-CF594 were added at 1:500 dilutions. Images were obtained with an EVOS FL imaging system (Thermo Fisher Scientific).

### 4.9. Mitochondrial Bioenergetics Assay

Mitochondrial bioenergetics assays were performed using a Seahorse XF analyzer (Agilent Technologies, Santa Clara, CA, USA) and following the manufacturer’s protocol. PE089 and Ire cells were seeded in the XF microplate with normal medium for 24 h. After cell attachment, cells were treated with or without EGF, gefitinib or AZD3965 (monocarboxylate transporter 1 inhibitor) for 24 h. The cell medium was replaced by medium without FBS and sodium bicarbonate (pH 7.4) and incubated at 37 °C without CO_2_ before analysis. The baseline measurements were recorded five times before injecting the following three compounds: oligomycin (2 μM), carbonylcyanide-3-chlorophenylhydrazone (CCCP, 6 μM) and rotenone (1.5 μM). The oxygen consumption rate (OCR), spare respiratory capacity, and proton leakage were automatically calculated and recorded using Seahorse XF24 software.

### 4.10. Mitochondrial Complex Activity Assay

Based on a protocol published by Salabei et al. [53], PE089 and Ire cells were seeded in the XF microplate with normal medium for 24 h. The mannitol and sucrose buffer (MAS buffer) were prepared with 70 mM sucrose, 220 mM mannitol, 10 mM KH_2_PO_4_, 5 mM MgCl_2_, 2 mM HEPES, 1 mM EGTA, and the pH value adjusted to 7.4 by 0.1 M KOH. Prior to the experiment, the MAS–BSA buffer was prepared with moderate fatty-acid free BSA (4 mg/mL). Before the experiment, cell media were replaced by MAS–BSA buffer with 1 nM PMP. For Complex I, we added 5 mM sodium pyruvate, 2.5 mM malate and 1 mM ADP as substrates. For Complex II, we treated permeabilized cells with 10 mM succinate, 1.5 μM rotenone and 1 mM ADP as the substrate. For Complex III, we measured the activity using 0.6 mM duroquinone and 1 mM ADP as the substrate. For Complex IV, we provided 2 mM ascorbate, 0.5 mM TMPD and 1 mM ADP as substrates. For Complex V, we added 1 mM ADP as the substrate. Then, injection B was 2 μM oligomycin. Injection C was 1.5 μM rotenone, 20 mM malonate, 2 μM antimycin A and 20 mM sodium azide. Finally, we calculated the respiratory control rate (RCR) as the output measurement (Supplementary Figure S2). The experiment was performed using a Seahorse XF24 analyzer.

### 4.11. Real-Time Quantitative RT-PCR

Total RNA was extracted by using NucleoZOL (Macherey-Nagel, Düren, Germany). Total DNA was extracted by the EasyPure Genomic DNA spin kit (Bioman, Taipei, Taiwan). RNA and DNA were quantified by Nanodrop 2000 (Thermo Fisher Scientific). RNA was reverse-transcribed to cDNA. An aliquot of 200 ng cDNA or 20 ng DNA was used as a template for each reaction and quantified by the StepOne Real-Time PCR System (Thermo Fisher Scientific). The primer sequences are shown in Table 2.

### 4.12. Statistical analysis

All the results were represented as means ± S.D. or S.E.M from three independent experiments and each experiment performed in duplicate. Statistical significances were determined by t-test analysis. A *p* value of < 0.05 was considered as statistically significant.

## 5. Conclusions

**Figure 8:**
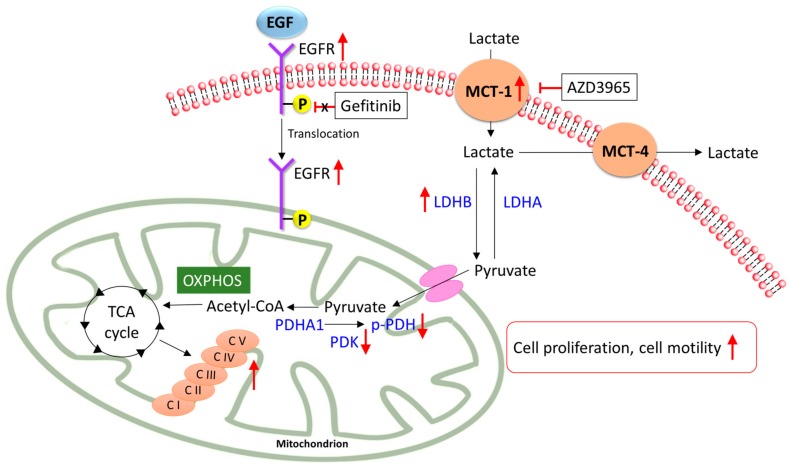
A schematic model of metabolic mechanisms in gefitinib-resistant Ire cells. A proposed model of the metabolic reprogramming that occurs during the development of TKI-resistant non-small cell lung cancer (NSCLC). In gefitinib-resistant NSCLC, EGFR translocated to mitochondria and strengthens mitochondrial oxidative phosphorylation (OXPHOS) capacity and complexes’ activity. Concomitantly, MCT-1, LDHB, and PDHA1 were highly expressed in gefitinib-resistant NSCLC. The inhibition of MCT-1 by AZD3965 reduced cell proliferation and cell motility in gefitinib-resistant NSCLC. Abbreviations: EGF, epidermal growth factor; EGFR, epidermal growth factor receptor; MCT, monocarboxylate transporter; LDH, lactate dehydrogenase; PDH, pyruvate dehydrogenase; PDK, pyruvate dehydrogenase kinase; C, complex; TCA cycle, tricarboxylic acid cycle; OXPHOS, oxidative phosphorylation.

In the present study, we investigated the different metabolisms of gefitinib-sensitive NSCLC and gefitinib-resistant NSCLC. We demonstrated that, during the development of gefitinib resistance, EGFR translocate to the mitochondria and strengthen the mitochondrial functions. This shifted the metabolism pathway from glycolysis to oxidative phosphorylation with increased expression levels of MCT-1, LDHB, and PDHA1 (Figure 8). Importantly, the gefitinib-resistant NSCLC, with a significantly elevated expression of MCT-1, presented a better response to the MCT-1 inhibitor, AZD3965. The treatment of AZD3965 effectively reduced the cell proliferation, cell viability, and cell motility of gefitinib-resistant NSCLC. In conclusion, our data suggest that inhibiting MCT-1 could be a potential strategy as a targeted therapy for non-small cell lung cancers, especially for gefitinib-resistant NSCLC.

## Figures and Tables

**Table 1:** Antibodies used in the study.

Antibody	Catalogue Number	Vendor
EGFR	sc-03	Santa Cruz Biotech
p-EGFR	#3777	Cell Signaling Technology
MCT-1	AB3538P	EMD Millipore
MCT-4	AB3316P	EMD Millipore
PDK-1α	ADI-KAP-PK112	Enzo Life Sciences
p-PDH	AP1062	EMD Millipore
LDHA	#2012	Cell Signaling Technology
LDHB	GTX101747	GeneTex
β-actin	GTX110564	GeneTex
COX IV	NB110-39115	Novus biologicals
MS604	ab110413	Abcam
HSP60	611562	BD Biosciences

**Table 2:** Oligonucleotide primers used for real-time quantitative RT-PCR.

Gene		Sequence (5′→3′)
*SCL16A1*	Forward	TGGATGGAGAGGAAGCTTTCTAAT
	Reverse	CACACCAGATTTTCCAGCTTTC
*SCL16A3*	Forward	GAGTTTGGGATCGGCTACAG
	Reverse	CGGTTCACGCACACACTG
*ACTB*	Forward	CCAACCGCGAGAAGATGA
	Reverse	CCAGAGGCGTACAGGGATAG
*LDHA*	Forward	AGCCCGATTCCGTTACC
	Reverse	CACCAGCAACATTCATTCCA
*LDHB*	Forward	CTAGATTTCGCTACCTTAT
	Reverse	TCATTGTCAGTTCCCATT
*PDHA1*	Forward	TGTGGAAGAACTAAAGGAAATTGATGT
	Reverse	TTCCAAAGGTGGCTCAGGAT
*PDK1*	Forward	CCGCTCTCCATGAAGCAGTT
	Reverse	TTGCCGCAGAAACATAAATGAG
*NDUFB8*	Forward	AGCCAGGTATTGACTGAATGTA
	Reverse	CACAGCACTGAGTTTTATTAGGGA
*SDHB*	Forward	GACACCAACCTCAATAAGGTCTC
	Reverse	GGCTCAATGGATTTGTACTGTGC
*UQCRC2*	Forward	CAAAGTTGCCCCCAAACTTA
	Reverse	AGCCATGTTTTCCCTTGTTG
*ATP5A*	Forward	GGTCAGCCGTCTCAGTCCATT
	Reverse	AACTAGCATCAACAGGTCCTC
*ND1*	L3441	ACTACAACCCTTCGCTGACG
	H3557	AGAAGAGCGATGGTGAGAGC
*CYTB*	L15260	AGTCCCACCCTCACACGATTC
	H15396	TTATCGGAATGGGAGGTGATTC
*COI*	L7075	GAGGCTTCATTCACTGATTTCC
	H7255	TTTCATGTGGTGTATGCATCG
*ATP6*	L8903	CCCACTTCTTACCACAAGGC
	H9059	GTGGCGCTTCCAATTAGGTG
*B2M*	Forward	CCAGCAGAGAATGGAAAGTCAA
	Reverse	CTCTCTCCATTCTTCAGTAAGTCAACT

## Supplementary Materials

The following are available online at https://www.mdpi.com/2072-6694/12/1/181/s1, Figure S1: A schema of the cellular bioenergetics parameters assessed by the XF24 extracellular flux analyzer, Figure S2: Index of mitochondrial complex activity. The schematic illustration represents the measurement of mitochondrial complex activity by Seahorse XF24 analyzer.
