# Supplementary material for: Inhibition of Alternative Cancer Cell Metabolism of EGFR Mutated Non-Small Cell Lung Cancer Serves as a Potential Therapeutic Strategy
Source: Cancers (Basel). 2020 Jan 10;12(1):181. doi: 10.3390/cancers12010181 (PMC7017237; doi:10.3390/cancers12010181)
Supplement: Supplementary file 1 [file cancers-12-00181-s001.pdf]

cancers

MDPI

Inhibition of Alternative Cancer Cell Metabolism of EGFR Mutated Non-Small Cell Lung Cancer Serves as a Potential Therapeutic Strategy

Chung-Yu Huang, Li-Han Hsu, Chung-Yeh Chen, Gee-Chen Chang, Hui-Wen Chang, Yi-Mei Hung, Ko-Jiunn Liu and Shu-Huei Kao

Supplementary Materials

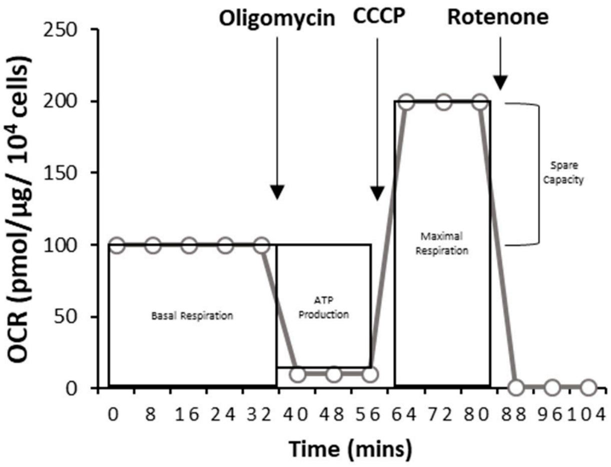

Figure S1. A schema of the cellular bioenergetics parameters assessed by the XF24 extracellular flux analyzer. The oxygen consumption rate (OCR) was measured under basal conditions, followed by the sequential addition of 2 μM oligomycin, 6 μM CCCP, and 1.5 μM rotenone. The progress curve is annotated to show the relative contribution of ATP-linked oxygen consumption, the maximal OCR after the addition of CCCP, the spare capacity, and the proton leak rate of cells.

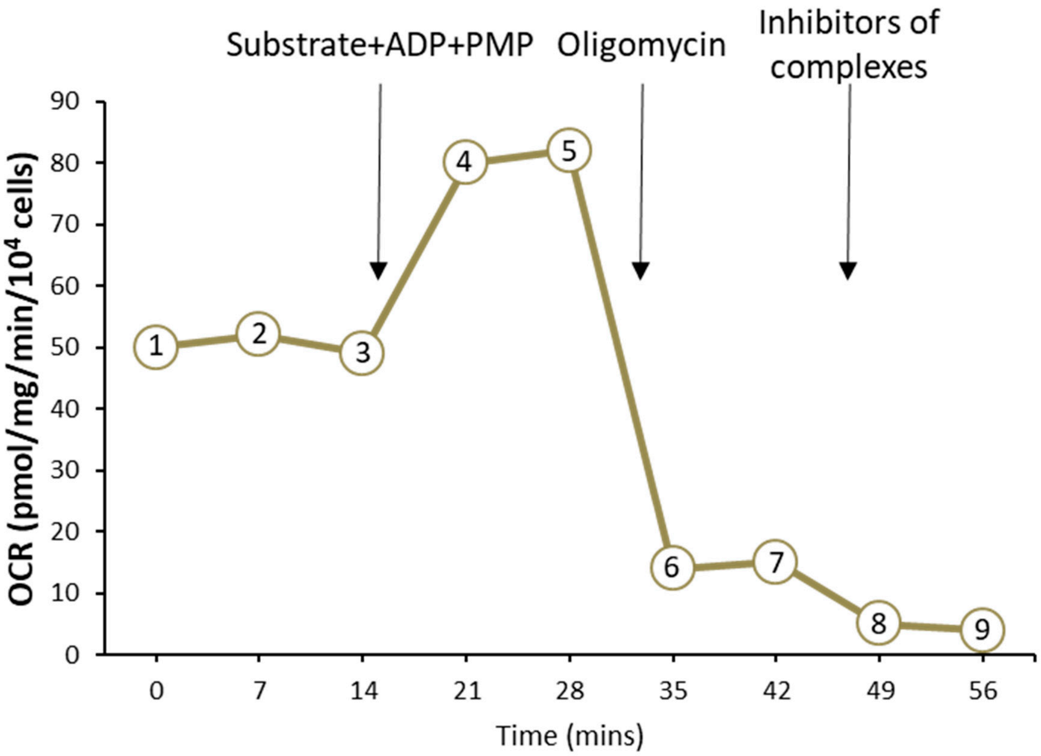

Figure S2. Index of mitochondrial complex activity. The schematic illustration represents the measurement of mitochondrial complex activity by Seahorse XF24 analyzer. Measurements 1 to 9 are shown here. After basal respiration, measurements (measurements 1 to 3) were recorded. Substrates with an ADP/PMP mixture were injected into wells to induce state 3 respiration. After 2 measurements (measurements 4 and 5), oligomycin was injected into wells to induce state 4 (measurements 6 and 7). Finally, inhibitors of mitochondrial complexes were injected into wells to inhibit mitochondrial respiration (measurements 8 and 9). State 3 OCR was calculated by measurements 4 minus measurements 8, which indicated that when substrates were added respiration increased and ATP was generated. After all the reagents were added and reacted, ADP was depleted, which was designated as state 4. State 4 OCR was calculated by measurements 6 minus measurements 8. State 3 divided by state 4 was the respiratory control rate (RCR). RCR was defined as the mitochondrial coupling state. A high RCR demonstrated a high capacity for substrate oxidation and ATP generation.
